# Fingerprint Detection and Differentiation of Gas-phase Amines Using a Fluorescent Sensor Array Assembled from Asymmetric Perylene Diimides

**DOI:** 10.1038/s41598-018-28556-x

**Published:** 2018-07-06

**Authors:** Yanyong Hu, Zichao Zhou, Feiping Zhao, Xiaoling Liu, Yanjun Gong, Wei Xiong, Mika Sillanpää

**Affiliations:** 1 Beijing National Laboratory for Molecular Sciences, Key Laboratory of Photochemistry, Institute of Chemistry, Chinese Academy of Sciences, Beijing, 100190 China; 2 University of Chinese Academy of Sciences, Beijing, 100049 China; 3 Laboratory of Green Chemistry, School of Engineering Science, Lappeenranta University of Technology, Sammonkatu 12, FI-50130 Mikkeli, Finland

## Abstract

A series of structurally analogous PDIs were fabricated and used as fluorescent sensor arrays. Adjustment of the molecular electron-donating ability and polarity (i.e., chemical structure) was found to greatly influence the fluorescent quenching by different types of amines. Moreover, the sensor array displayed high sensitivity to amine vapors and allowed the fingerprint differentiation of different species.

## Introduction

Optical sensors, especially fluorescent detectors, demonstrate the rapid, practical, and sensitive identification of an extensive range of organic vapors^1^. Great efforts in this field have concentrated on the detection of trace analytes and lowering the existing detection limits^2–4^. Accompanied by the long-term development and research history of optical probes^5,6^, sensor arrays that can quickly detect and accurately discriminate target analytes are highly desirable and have practical significance in a variety of fields^7^, for example, medical diagnosis^8,9^, environmental monitoring^10^, health^11^ and so on.

Research into array sensing initially focused on the detection and differentiation of diverse vapors with different chemical properties to simulate the olfactory system in mammals^12,13^. In 2000, Prof. Suslick and co-workers firstly reported a facile method based on optical probes to identify and distinguish different classes of volatile organic compounds (VOCs)^14^. Since then, a series of optical sensor arrays have been developed through the rational adjustment of composition and structure of the array^15^. These reported sensory devices have led to the precise identification of specific analytes from chemical compounds and the discrimination of analytes with similar chemical properties^16,17^. In recent years, Prof. Swager and co-workers developed a sequence of approaches, including electric sensors (e.g., chemiresistive devices)^18^, ^19^F NMR receptors^19^ and fluorescence techniques^20,21^, to selectively detect and differentiate VOCs, especially neutral organic compounds^22^, explosives and common amines. To further lower the achieved detection limits and realize the fingerprint identification of amine vapors, we investigated the detection and differentiation performance of fluorescent sensor arrays assembled from perylene diimide molecules (PDIs) with a simple detection method. Using a dual-mode responsive sensory material (i.e., nanoribbons with fluorescent and photoconductive features)^23^, our group succeeded in selectively discriminating alkyl amines from aromatic amines, but the distinction among different alkyl amines and aromatic amines was not successful. Herein, we demonstrate that using fluorescent sensor arrays assembled from structurally analogous PDIs allows for the fingerprint detection and differentiation of different types of trace amine vapors.

## Results and Discussion

**Figure 1:**
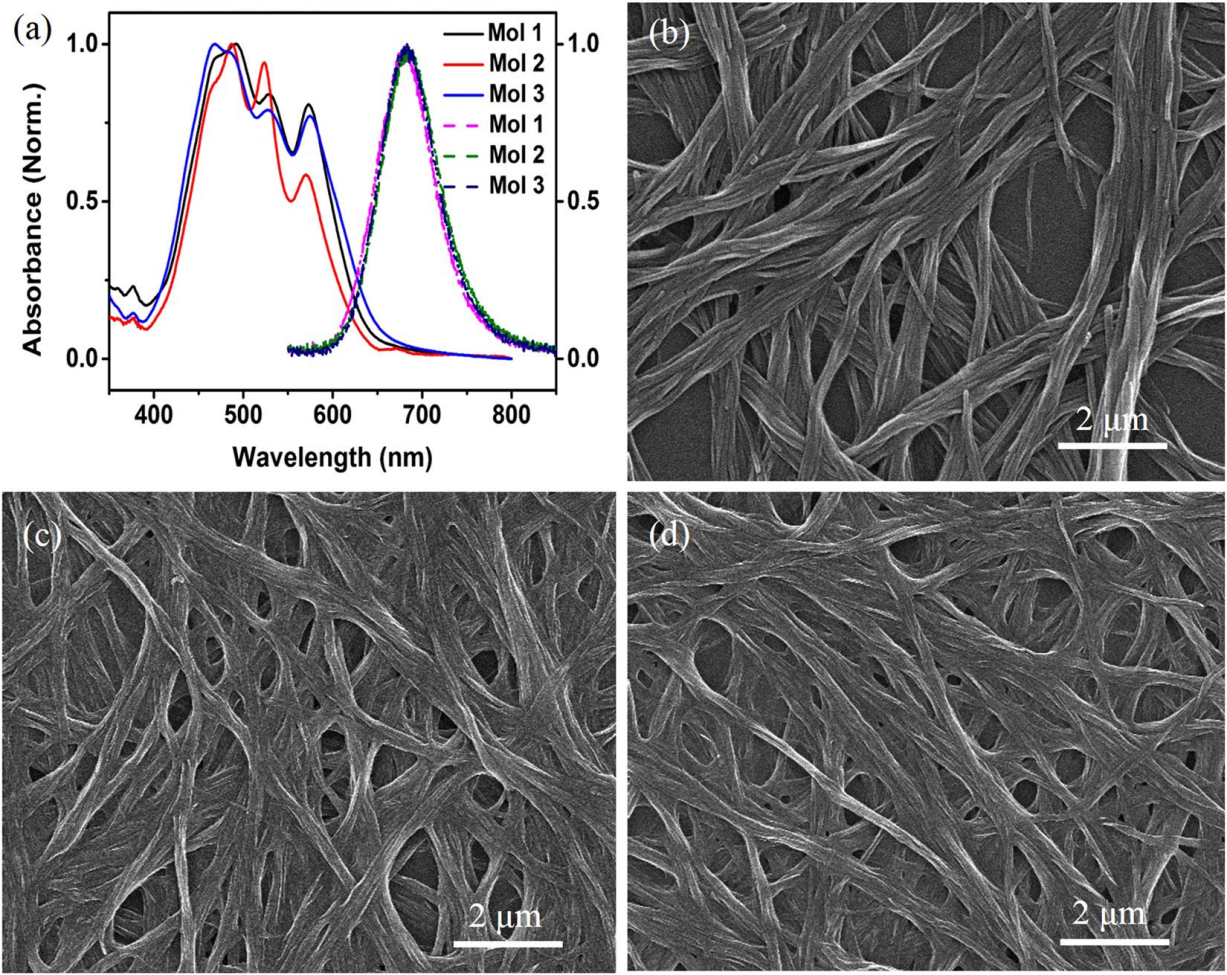
(**a**) Absorption spectra (solid lines) and fluorescent profiles (dashed lines) of the nanoribbons assembled from PDI **1–3**. (**b**) Typical SEM image of **AAM-1**. (**c**) Typical SEM image of **AAM-2**. (**d**) Typical SEM image of **AAM-3**.

Motivated by previous work^23^, we selected the asymmetric perylene diimide molecule **1** as the primitive molecule, which has a methoxy substituent at the 4-position of the phenyl moiety at one end and dodecyl at the other (Figure [S1a]). The relevant synthesis and self-assembly of molecule **1** followed reported processes^24–26^, and the obtained morphology (aggregates assembled from molecule **1**: **AAM-1**) is presented in Fig. [1b]. It’s well known that chemical properties especially abilities of electron-donation and polarity are different for hydroxyl group, mercapto group and methoxy group (e.g. the ability of electron-donating and polarity decreased). To achieve a fluorescent PDI-based sensory array capable of sensitively detecting and clearly discriminating selected amines, we adjusted the electron-donating ability and polarity (i.e., chemical structure) of PDIs by tuning specific end groups. To this end, we successfully fabricated molecule **2** (with a mercapto substituent at the 4-position of the phenyl moiety at one end and dodecyl at the other) and molecule **3** (with a hydroxyl substituent at the 4-position of the phenyl moiety at one end and dodecyl at the other) (Figure [S1a]), the synthetic details were demonstrated in the methods. Molecules **2** and **3** were assembled under identical conditions as **AAM-1** to obtain the relevant aggregates (i.e., **AAM-2** and **AAM-3**). Typically, 0.3 mL of the monomer of a specific molecule was dissolved in chloroform solution (to obtain 0.1 mM molecule **1**, 0.1 mM molecule **2**, or 0.05 mM molecule **3**) and injected into 4.5 mL ethanol. Then, the obtained mixtures were completely mixed and aged at room temperature for 3 days. The resulting morphologies of **AAM-2** and **AAM-3** were characterized by a Hitachi S-4800 scanning electron microscope (SEM) operating at 10 kV, as depicted in Fig. [1c,d], revealing that morphologies of the three aggregates are analogous regardless of their specific chemical structure at side chains. However these aggregates exhibit significantly different fluorescence quantum yield (FQY). FQY of **AAM-1** exhibited 35 ± 0.4%, while that of **AAM-2** and **AAM-3** were 14 ± 0.3% and 13 ± 0.3% respectively. Figure [1a] displays the optical properties of the three aggregates (optical properties of the respective monomers are shown in Figure [S1b]), and optical properties of aggregates (e.g. absorbance and fluorescence spectra) contributed by dried aggregates. Evidently, there is little difference in the absorption spectra, as shown by the similar absorption bands at 464–475, 485–494, 552–530, and 571–575 nm. Furthermore, the characteristic emission maxima were similar, ranging from 680 to 685 nm. Considering the above results, the applied organizational behavior of the three PDIs during the self-assembly process can be concluded to be similar.

**Figure 2:**
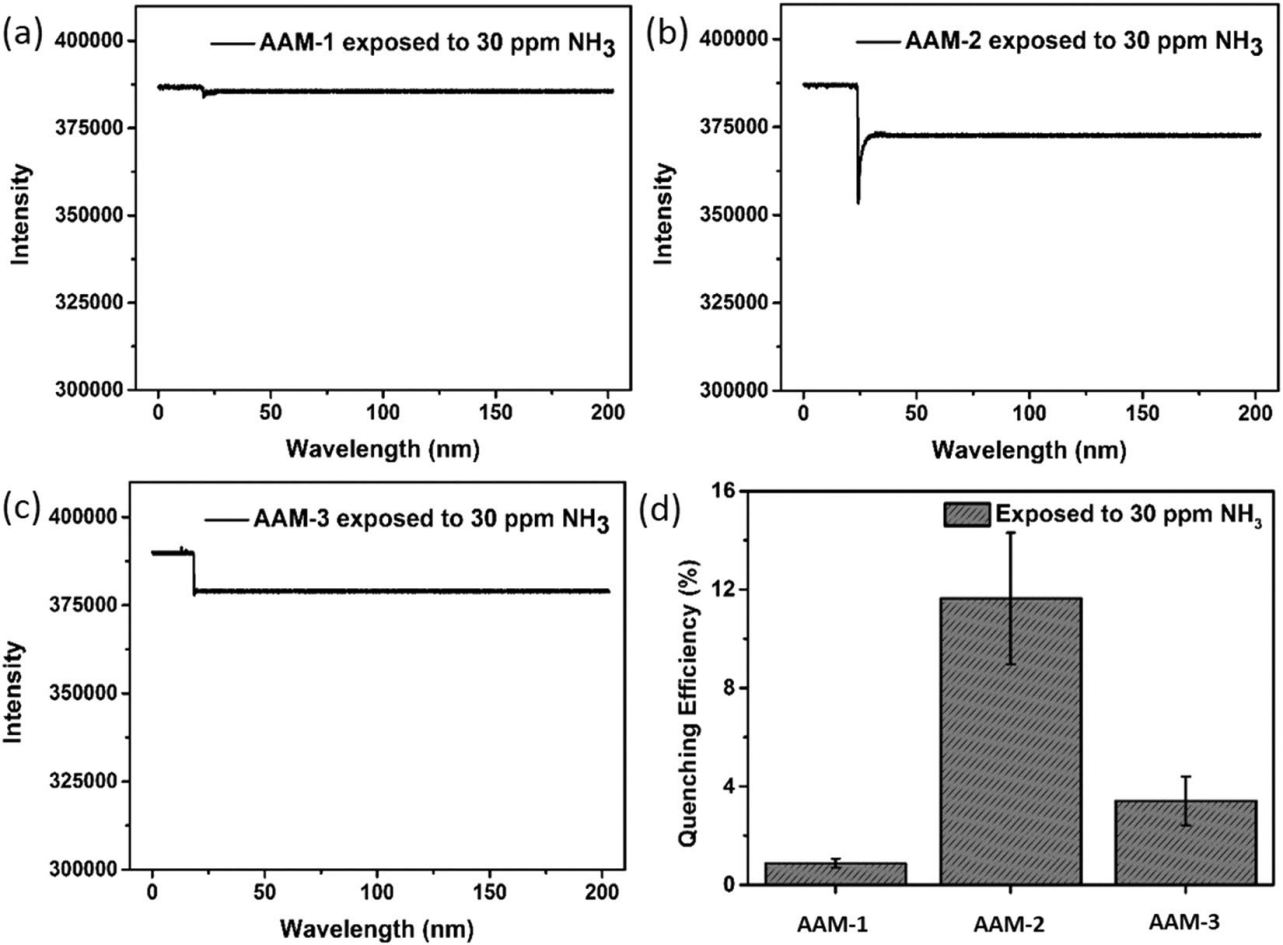
Typical time-dependent fluorescence quenching spectra of (**a**) **AAM-1**, (**b**) **AAM-2** and (**c**) **AAM-3** upon exposure to 30 ppm NH_3_ vapor. (**d**) Fluorescence quenching efficiency of **AAM-1**, **AAM-2** and **AAM-3** upon exposure to 30 ppm NH_3_ vapor.

**Figure 3:**
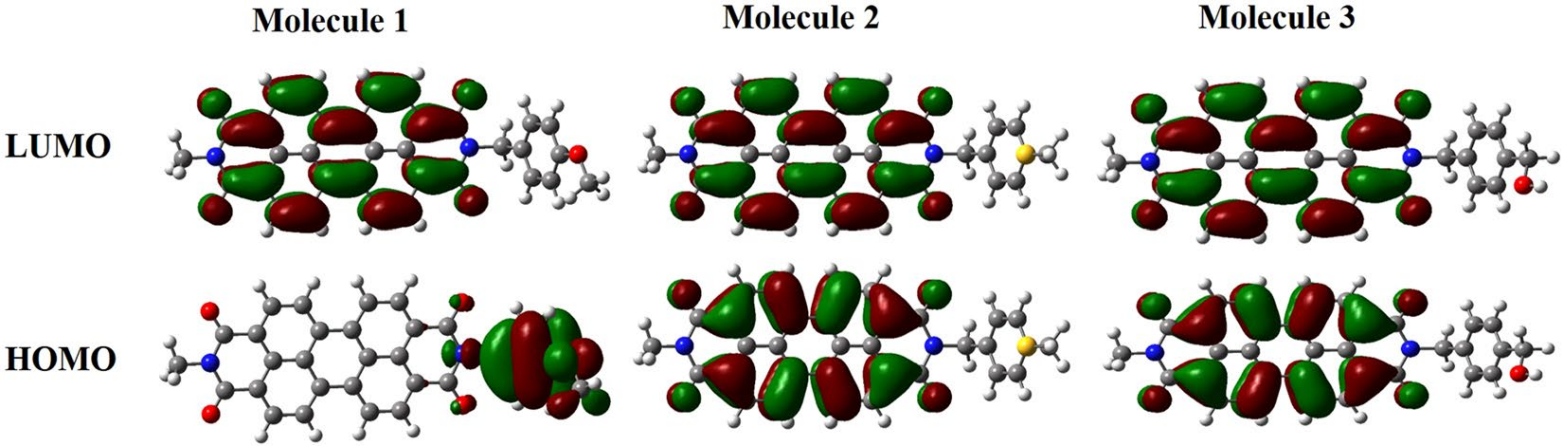
The lowest unoccupied molecular orbital (LUMO) and highest occupied molecular orbital (HOMO) of molecule **1**, molecule **2** and molecule **3** calculated with geometry optimization by the B3LYP/6–31 G* method.

To gain insight into the response of the fluorescent nanoribbons upon the introduction of volatile amine vapors, sensing experiments were conducted with a homemade optical chamber based on an Ocean Optics USB4000 device^27^. As demonstrated in Fig. [2a], when 30 ppm NH_3_ was pumped onto **AAM-1** nanoribbons, the recorded fluorescence intensity slightly reduced by 0.88 ± 0.19%. However, exposure to 30 ppm NH_3_ quenched the fluorescence of **AAM-2** by 11.64 ± 2.67% (Fig. [2b]). By contrast, for **AAM-3** (Fig. [2c]), the fluorescence was quenched by 3.41 ± 1%. The obtained results (Fig. [2d]) suggest that among the three selected materials, **AAM-2** is most responsive to NH_3_ under the same operating conditions. Considering the identical structure of the PDI core, we calculated the lowest unoccupied molecular orbital (LUMO) and highest occupied molecular orbital (HOMO) of molecule **1**, molecule **2** and molecule **3** with geometry optimization by the B3LYP/6–31 G* method (details were shown in the methods). As depicted in Fig. [3], the electron density of the HOMO for molecule **1** was distributed on the end group that have methoxy substituted at the 4-position of the phenyl moiety, however the electron density of the HOMO for molecule **2** and molecule **3** were distributed on the PDI core. Besides, on account of different chemical structure (e.g. mercapto group and hydroxyl group respectively), the electron density of the PDI core for molecule **2** and molecule **3** were slightly different. It’s obvious that electron density of the HOMO for molecule **1**, molecule **2** and molecule **3** were distinct, while that of LUMO for three molecules were the same, which were localized on the orbitals of the PDI core. The difference of the three sensor members in fluorescent response ability to NH_3_ is likely results from the difference in electron density attributed by chemical structure.

Fluorescent responses of three sensor members (e.g. **AAM-1**, **AAM-2** and **AAM-3**) to other six kinds of common amine vapors at the concentration of 30 ppm were then explored. As can be seen from Figure [S2], three sensor members exhibit different responses toward all these amine vapors. According to our previous report^4,28^, the fluorescence quenching mechanism was mainly due to photoinduced electron transfer (PET process), by which electrons were transferred from amines to the excited PDIs. The difference in driving forces (Figure [S3]) played a critical role in the obtained fluorescence response^29^. During the PET process, the electron-accepting abilities of sensor members (e.g. **AAM-1**, **AAM-2** and **AAM-3**) were not the same, and the electron-donating ability of seven classes of amines were different, both of that making the fluorescence quenching of each sensor member to amines were different. It’s observed that the sensory responses were irreversible in air (Fig. [2] and Figure [S2]) on account that adsorption process existed during the strong physicochemical interaction between amine molecules and the sensory materials. However, after heating the sensory materials at 60 °C for 1 hour, the fluorescence intensity was recovered, making the sensory array could be reused (Figure [S4]).

**Figure 4:**
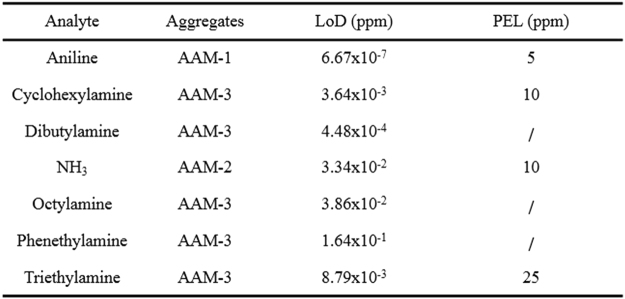
Detection limits of seven selected amines calculated from the responses of the sensor arrays and the recommended safety limits.

**Figure 5:**
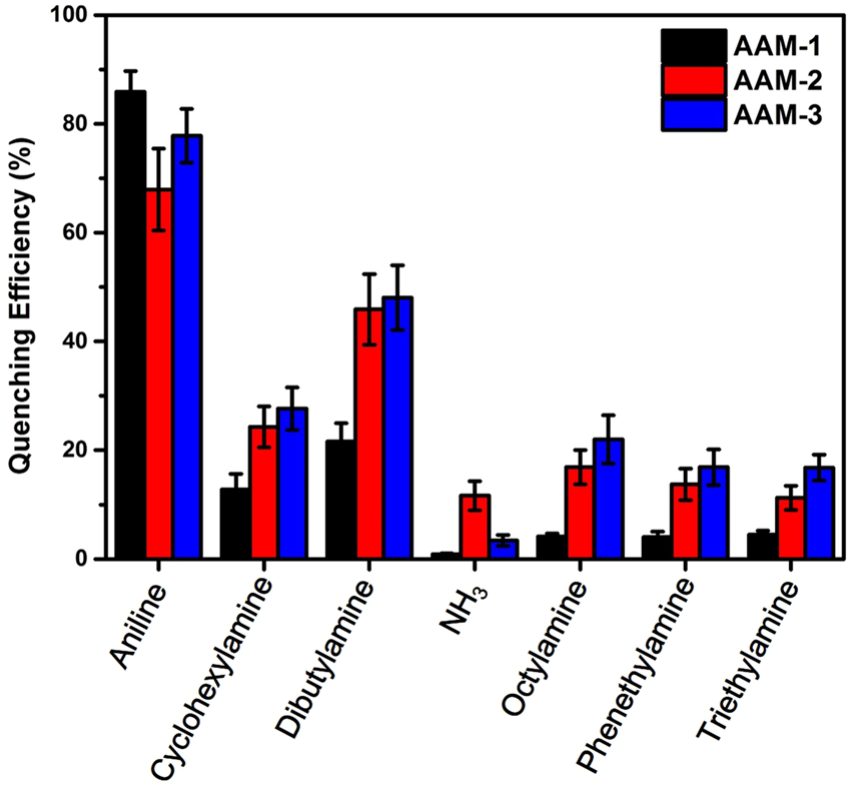
Fluorescence responses of the sensor arrays constituted of **AAM-1**, **AAM-2** and **AAM-3** to seven selected amine vapors at concentrations of 30 ppm.

Inspired by these initial investigations, we further explored the utility of the three aggregates as a fluorescent sensor array. Seven types of industrially common amines were selected (listed in Fig. [4]), and the fluorescent quenching effects of each amine on different nanoribbons were evaluated under ambient conditions. Amine vapors at concentration of 30 ppm were blown onto the three materials, and the detailed processing patterns and experimental setups were described in the methods. To ensure the accuracy and authenticity of sensor data, each experiment was carried out twelve times. By removing the highest and lowest values, the obtained average value was used for the comprehensive evaluation of the sensor array. Note that no correction, selection or pre-processing of the data was performed, and the calculated quenching responses are shown in Fig. [5]. Obviously, the three sensor members show different responses to each amine. **AAM-2** was found to be the most suited for the detection of NH_3_. By comparison, **AAM-1** demonstrated superior performance in response to aniline, whereas **AAM-3** was the best for detecting the other five selected amines. Interestingly, the fluorescence quenching of the sensor material upon exposure to amine vapors at a lower concentration (3 ppm) is consistent with that at 30 ppm, as seen in Figure [S5]. To explore the fluorescent responses of three sensor members (e.g. **AAM-1**, **AAM-2** and **AAM-3**) under different humidity levels, we tested fluorescent experiment to water vapor with the same method. As can be seen from Figure [S6], when saturated water vapor (31280 ppm) were pumped onto **AAM-1**, only a small pose of fluorescence quenching appeared. Besides, without any treatment the small pose can recover immediately. When water vapor at the concentration of 3128 ppm were pumped onto **AAM-1**, the fluorescence intensity didn’t change. The same phenomenon occurred when the sensor member was replaced by **AAM-2** and **AAM-3** respectively. On account that the usual humidity range in real life is within 20–70% (water vapor concentration vary from 6256 ppm to 21896 ppm), the concentration are within the concentration tested (e.g. 3128 ppm and 31280 ppm). Therefore, sensor performances of sensor members were free from the changes of humidity levels.

**Figure 6:**
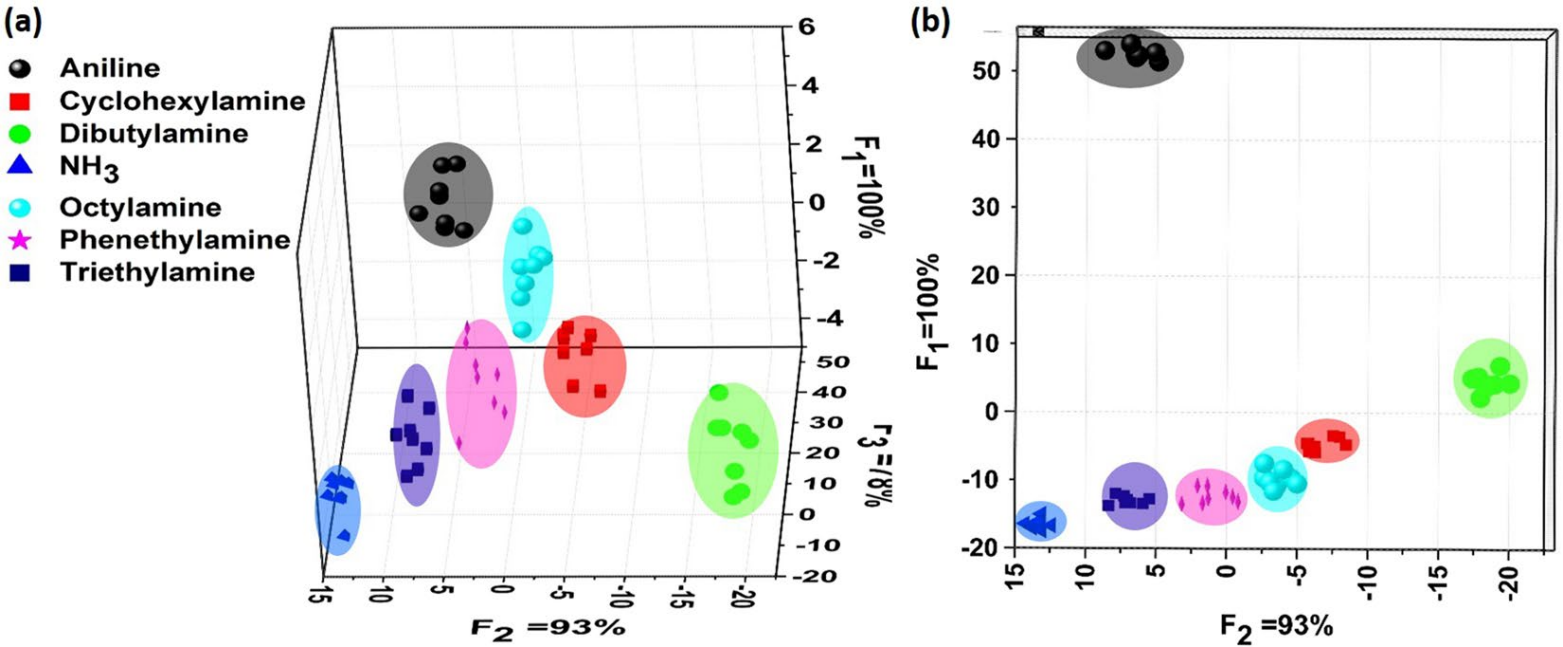
Discriminant analysis of a fluorescent sensor array composed of aggregates of **AAM-1**, **AAM-2** and **AAM-3**. (**a**) Three-dimensional LDA graph and (**b**) two-dimensional LDA graph displaying the distinct clustering of seven selected amines (30 ppm).

It’s reported that linear discriminant analysis (LDA) is a supervised technique for dimensionality reduction that tries to represent the total data in minimum dimension and also make sure that different classes can be differentiated (classification)^30^. Considering the dramatically different fluorescence responses and different types of amines, the LDA statistical method was used quantitatively to investigate the similarities of fluorescence responses and to cluster the obtained data with the aim to differentiate analytes^8,20,31^. Different features of the recorded time-dependent fluorescence quenching spectra (as shown in Fig. [2] and Figure [S2]) demonstrated important information to classify analytes. To minimize deviation caused by LDA classification^20^, 10 data points from each of 10 recorded time-dependent fluorescence quenching spectra (used to calculated quenching responses of Fig. [5] as described in the paragraph before this one) were used to perform LDA analysis. These data points were measured at 5 time points, and two quenching intensities were used at every time point. The 5 time points were T_0_ (at the beginning of exposure), T_1_ (18 s), T_2_ (35 s), T_3_ (50 s) and T_4_ (100 s) respectively. Figure [6] presents the LDA results of the sensor arrays, and the concentration of amine vapors were 30 ppm. Figure [6] shows the best subspace that correctly classify the fluorescence data obtained. Parameters F_1_, F_2_ and F_3_ represent the standard deviation along the x, y and z axis respectively, and the meaning of percentage is the proportion of contribution resulted from the square roots for the diagonal elements of data covariance matrix^32^. These analytes were classified perfectly under the experiment condition of our fluorescent procedure, with close to 100% discrimination and classification (Fig. [6] and Figure [S7]). Unfortunately, these analytes could not be distinguished at the concentration of 3 ppm (Figure [S8]), and the discriminatory limit of the PDI sensor array under our experiment conditions was 30 ppm.

To better assess the fluorescence response of the sensor array, responses of the nanoribbons to amines at different concentrations were recorded (testing concentrations were listed in Figure [S8]). To obtain relevant detection limits to different amines using the nanoribbons^4^, the fluorescence quenching data were fitted to the Langmuir equation, as displayed in Figure [S10]. The detection limits to the selected analytes, considering the fluorescence quenching efficiency and background noise, are summarized in Fig. [4], which further reflect that **AAM-1**, **AAM-2** and **AAM-3** exhibit different sensitivities to amines. Notably, the detection limits of aniline reached the ppt level (6.67 × 10^−1^ ppt), and those of the other five amines were decreased to the ppb range, except for that of phenethylamine (1.64 × 10^−1^ ppm). The detection limits of the other five amines (i.e., cyclohexylamine, dibutylamine, NH_3_, octylamine, and triethylamine) were found to be 3.64 ppb, 4.48 × 10^−1^ ppb, 33.4 ppb, 38.6 ppb, and 8.79 ppb, respectively. Therefore, the superior sensitivity to the selected amines, which are below the exposure limit set by permissible exposure limit (PEL)^14^, demonstrate the excellent performance of the developed fluorescent sensor array^20^.

In summary, fluorescent PDI-based sensory arrays constituted of aggregates assembled from structurally analogous PDIs were developed in this work. By adjusting the molecular electron-donating ability and polarity (i.e., chemical structure) by tuning the specific end groups of PDI, the obtained aggregates exhibited different sensitivities to a variety of amines, which made it possible to realize the fingerprint detection and selective differentiation of different types of trace amine vapors in the ppm range. Furthermore, the sensors exhibit distinct high sensitivity to the selected amines. The calculated detection limit toward aniline reached the ppt level, and that of the other amines reached the ppb level, except for phenethylamine, proving the sensor array to be a good candidate for a gas monitor.

## Methods

### Synthesis procedures

#### Synthesis procedure of molecule 2

Compound **2**: A solution of **1** (2 g, 10.2 mmol) was dissolved in 15 mL of THF, then 13.26 mmol of HSAc and 13.26 mmol of anhydrous K_2_CO_3_ were added to the above solution under the protection of Ar. The mixture was stirred at room temperature in an air-free flask for 1.5 h, and the completion of the reactions was monitored by thin-layer chromatography (TLC). Then THF was removed by rotary evaporation and the residue was extracted with ethyl acetate (3 × 20 mL). The combined organic layers were dried by sodium sulfate, and concentrated under vacuum. The crude product obtained was sufficiently pure to use directly in the next step without any further purification.

Compound **2** ^1^HNMR (400 MHz, CDCl_3_) δ 7.59–7.57 (d, *J* = 8.0 Hz, 2 H), 7.41–7.39 (d, *J* = 8 Hz, 2 H), 4.124(s, 2 H), 2.397 (s, 3 H).

Compound **3**: LiAlH4 (954 mg, 25.12 mmol) was slowly added to a stirring solution of 15 mL THF under 0 °C, then the suspension was stirred for 10 min at room temperature. A solution of compound **2** (1.2 g, 6.28 mmol) in THF (5 mL) was added dropwise to the suspension solution. The mixture was refluxed for 2 h under Ar. After cooling to room temperature, the reaction was quenched by 5 mL 1 N HCl solution, and stirred for another 30 min. After filtration, the organic solution was concentrated under vacuum to afford compound **3**.

Compound **3** ^1^HNMR (400 MHz, DMSO-*d6*) δ 8.24 (br, 2 H), 7.41–7.36 (m, 4 H), 3.99 (s, 2 H), 3.75–3.73 (d, *J* = 8.0 Hz, 2 H), 2.87–2.83 (t, *J* = 8.0 Hz 1 H).

Compound **5**: A solution of **4** (200 mg) and dodecanamine (1 g) in MeOH (30 ml) was refluxed for 7 hours under N_2_. After cooling to room temperature the solvent was added con HCl (20 ml) and stirred overnight. The resulting red solid was collected by vacuum filtration through a 0.45 μm membrane filter, rinsed thoroughly with water and ethanol, and then concentrated under vacuum to afford compound **5** which was not purified and was used directly for the following reaction.

Molecule **2**: A mixture of **5** (30 mg) and **3** (100 mg) in imidazole (2 g) was stirred for 1 hour at 140 °C under Ar. After cooling to room temperature, 10 mL 1 N HCl solution were added to the mixture and stirred for 30 min. The resulting red solid was collected by vacuum filtration through a 0.45 μm membrane filter. The residue was purified by column chromatography (silica, chloroform: acetone = 100:0.2) to afford molecule **2**. The specific synthesis procedures of molecule **2** are shown in Figure [S11].

Molecule **2** ^1^HNMR (400 MHz, CDCl_3_) δ 8.72–8.64 (m, 8 H), 7.55-7.53 (d, *J* = 8.0 Hz 2 H), 7.29-7.27 (d, *J* = 8.0 Hz, 2 H), 5.40 (s, 2 H), 4.21 (t, *J* = 8.0 Hz, 2 H), 3.71 (d, *J* = 8 Hz, 2 H), 1.74 (m, 2 H), 1.71 (t, *J* = 8.0 Hz, 6 H), 1.32-1.25 (m, 18 H), 0.90-0.85 (m, 3 H). MALDI-MS: (m/z) = 694.7. ^1^HNMR and MALDI-MS of Molecule **2** are shown in Figure [S12] and Figure [S13] respectively.

#### Synthesis procedure of molecule 3

Compound **2**: A solution of **1** (200 mg) and dodecanamine (1 g) in MeOH (30 ml) was refluxed for 7 hours under N_2_. After cooling to room temperature the solvent was added con HCl (20 ml) and stirred overnight. The resulting red solid was collected by vacuum filtration through a 0.45 μm membrane filter, rinsed thoroughly with water and ethanol, and then concentrated under vacuum to afford **2** which was not purified and was used directly for the following reaction.

Molecule **3**: A mixture of **2** (20 mg), (4-(aminomethyl)phenyl)methanol (20 mg) in imidazole (2 g) and zinc acetate (2 mg) was stirred for 3 hours at 140 °C under N_2_. After cooling to room temperature, 1 N HCl solution (100 mL) were added to the mixture and stirred for 2 hours. The resulting red solid was collected by vacuum filtration through a 0.45 μm membrane filter and rinsed thoroughly with water and ethanol. The residue was purified by column chromatography (silica, chloroform: acetone = 20:1) to afford the product and recrystallized from ethanol. The specific synthesis procedures of molecule **3** are shown in Figure [S14].

Molecule **3** ^1^HNMR (400 MHz, CDCl_3_) δ 8.72-8.64 (m, 8 H), 7.59-7.57 (d, *J* = 8.0 Hz, 2 H), 7.35-7.33 (d, *J* = 8.0 Hz, 2 H), 5.42 (s, 2 H), 4.67 (d, *J* = 4 Hz, 2 H), 4.21 (t, *J* = 8 Hz, 2 H), 1.76 (m, 2 H), 1.32-1.25 (m, 18 H), 0.89-0.84(m, 3 H). MALDI-MS: (m/z) = 678.2. ^1^HNMR and MALDI-MS of Molecule **3** are shown in Figure [S15] and Figure [S16] respectively.

### Fluorescence sensing measurements of amines

The fluorescence quantum yields of the aggregates (**AAM-1**, **AAM-2** and **AAM-3**) deposited on a polytetrafluoroethene (PTFE) film were determined by an integrating sphere method that was performed on Hamamatsu Absolute PL Quantum Yieldspectrometer C11247. The fluorescence quenching response of the nanoribbons by amines were measured with the method similar to the previously reported^27^. The fluorescence quenching responses of the aggregated nanoribbons to amine vapors were examined on a home-built detector^1^. A jar (100 mL) containing 5 ml of solvent was sealed for 24 h to obtain saturated amine vapors. And some cotton was placed above the solvent in the jar to keep the vapor pressure at a constant value. By injecting a small volume of saturated vapor of a specific amine into a sealed vial (100 mL), amines at a required diluted vapor concentration are achieved. For insistence, injecting 100 μL saturated aniline vapor (831 ppm) into a 100 mL vial will produce a vapor concentration with 1000 times diluted, i.e., 0.831 ppm^4^.The changes of fluorescence spectra and time-dependent fluorescence profile were recorded with an Ocean Optics USB4000 fluorometer using a 455 nm LED lamp as the light source. The fluorescence response was carried out by blowing 3 mL vapors at certain concentration into a quartz tube and the vapors would be pumped onto the nanoribbons in the quartz tube by an air pump. The flow rate was set at 125 mL/min. While the emission was continuously recorded by the fluorometer.

### SEM measurements of the sensor materials

Typically, 0.3 mL of the monomer of a specific molecule was dissolved in chloroform solution (to obtain 0.1 mM molecule **1**, 0.1 mM molecule **2**, or 0.05 mM molecule **3**) and injected into 4.5 mL ethanol. Then, the obtained mixtures were completely mixed and aged at room temperature for 3 days. The resulting morphologies of **AAM-1**, **AAM-2** and **AAM-3** were characterized by a Hitachi S-4800 scanning electron microscope (SEM) operating at 10 kV. Following was the process for proceeding from the solution of the molecules to a sample for SEM analysis: The suspending aggregates in solution were drop-casted onto a silica substrate respectively, and then Pt were sputtered on the surface by a LEICA EM SCD-500 (The sputtered Pt layer was ca. 3 nm).

### Theoretical calculations of the LUMO and HOMO

Theoretical calculations of the lowest unoccupied molecular orbital (LUMO) and highest occupied molecular orbital (HOMO) of molecule **1**, molecule **2**, molecule **3** and amines were performed using Gaussian 09 suite of programs^33^. Computational strategy was relied on DFT to optimize LUMO and HOMO of the ground state of molecule **1**, molecule **2**, molecule **3** and amines. The ground-state geometry of analytes were optimized using the B3LYP/6-31 G* atomic basis set at the DFT level.

## Electronic supplementary material

Dataset 1
