# Supplementary material for: Fingerprint Detection and Differentiation of Gas-phase Amines Using a Fluorescent Sensor Array Assembled from Asymmetric Perylene Diimides
Source: Sci Rep. 2018 Jul 6;8:10277. doi: 10.1038/s41598-018-28556-x (PMC6035276; doi:10.1038/s41598-018-28556-x)
Supplement: Supplementary file 1 — Dataset 1 [file 41598_2018_28556_MOESM1_ESM.docx]

Supporting Information

**Fingerprint Detection and Differentiation of Gas-phase Amines Using a Fluorescent Sensor Array Assembled from Asymmetric Perylene Diimides**

Yanyong Hu, ^*1,2^ Zichao Zhou, ^1,2^ Feiping Zhao, ^3^ Xiaoling Liu, ^1,2^ Yanjun Gong, ^1,2^ Wei Xiong, ^1,2^ Mika Sillanpää ^*3^

^1^Beijing National Laboratory for Molecular Sciences, Key Laboratory of Photochemistry, Institute of Chemistry, Chinese Academy of Sciences, Beijing 100190, China;

^2^University of Chinese Academy of Sciences, Beijing 100049, China;

^3^Laboratory of Green Chemistry, School of Engineering Science, Lappeenranta University of Technology, Sammonkatu 12, FI-50130 Mikkeli, Finland.

*Corresponding author.

Yanyong Hu. (email: huyanyong77@iccas.ac.cn) and Mika Sillanpää (email: Mika.Sillanpaa@lut.fi).

**ABSTRACT:** A series of structurally analogous PDIs were fabricated and used as fluorescent sensor arrays. Adjustment of the molecular electron-donating ability and polarity (i.e., chemical structure) was found to greatly influence the fluorescent quenching by different types of amines. Moreover, the sensor array displayed high sensitivity to amine vapors and allowed the fingerprint differentiation of different species.

Table of Contents

1. Other supporting figures

1) Figure [S1] ...................................................................................................... S-2

2) Figure [S2] ...................................................................................................... S-3

3) Figure [S3] ...................................................................................................... S-4

4) Figure [S4] ...................................................................................................... S-5

5) Figure [S5] and Figure [S6]............................................................................ S-6

6) Figure [S7] and Figure [S8]............................................................................ S-7

6) Figure [S9] ...................................................................................................... S-8

6) Figure [S10] and Figure [S11]............................................................................ S-9

6) Figure [S12] and Figure [S13]............................................................................ S-10

6) Figure [S14] and Figure [S15]............................................................................ S-11

6) Figure [S16] ....................................................................................................... S-12

**1. Other supporting figures.**


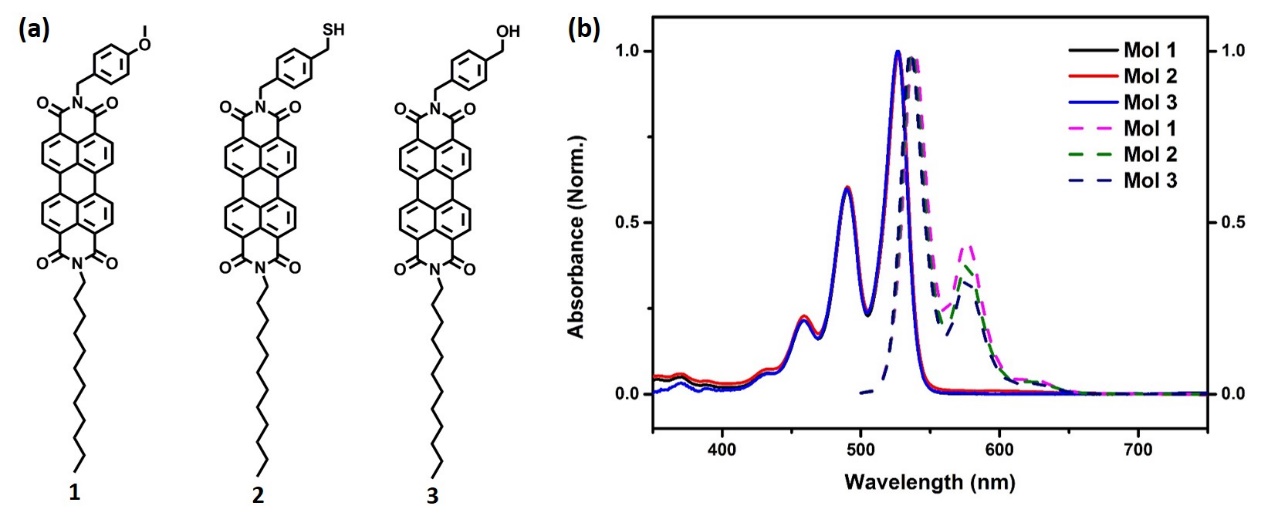


Figure [S1]. (a) Molecular structures of PDI **1-3**. (b) Typically absorbance (solid lines) and fluorescence spectra (dashed lines) of monomer **1-3**.


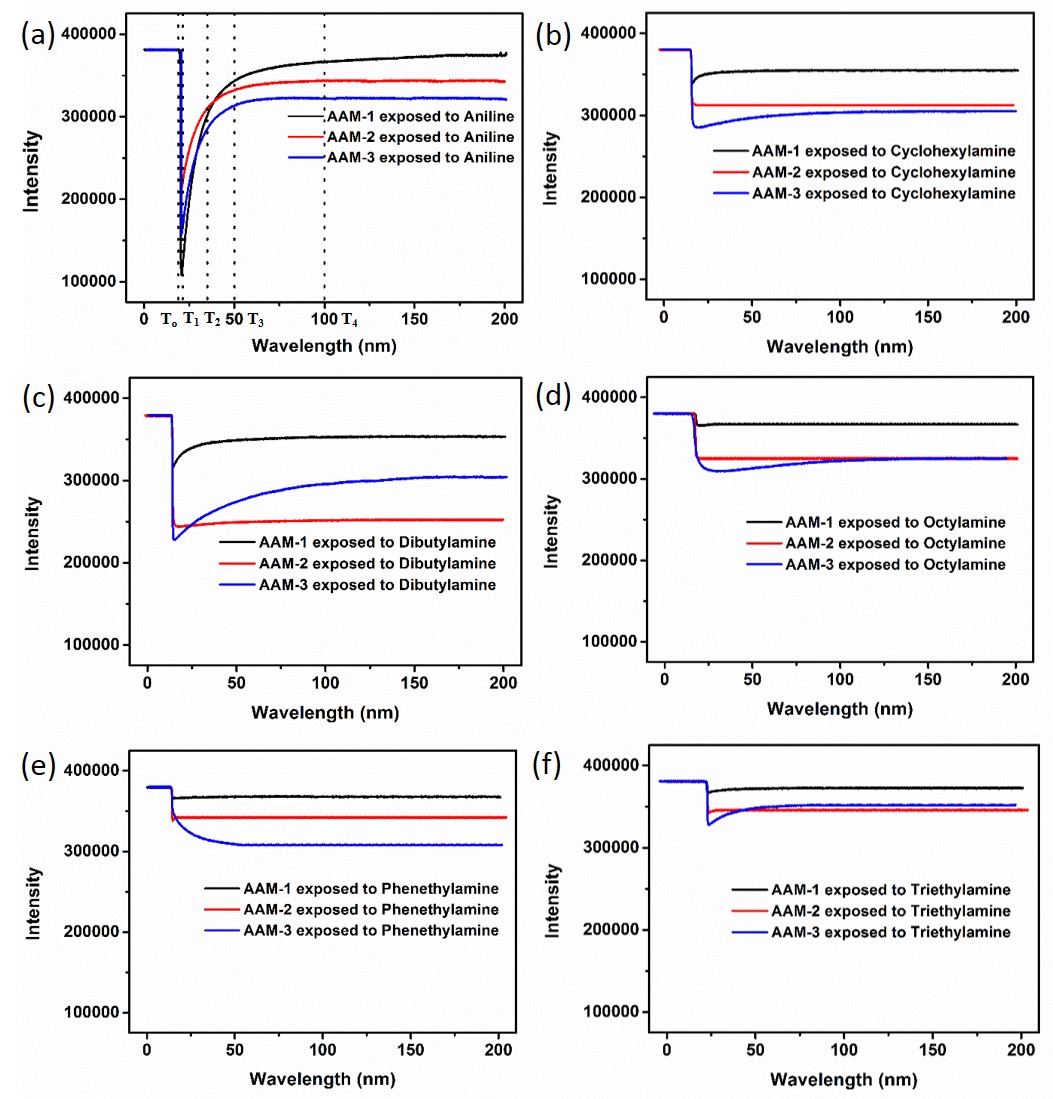
s

Figure [S2]. Typical time-dependent fluorescence quenching spectra of **AAM-1** (Black), **AAM-2** (Red) and **AAM-3** (Blue) upon blowing 30 ppm amine vapors.


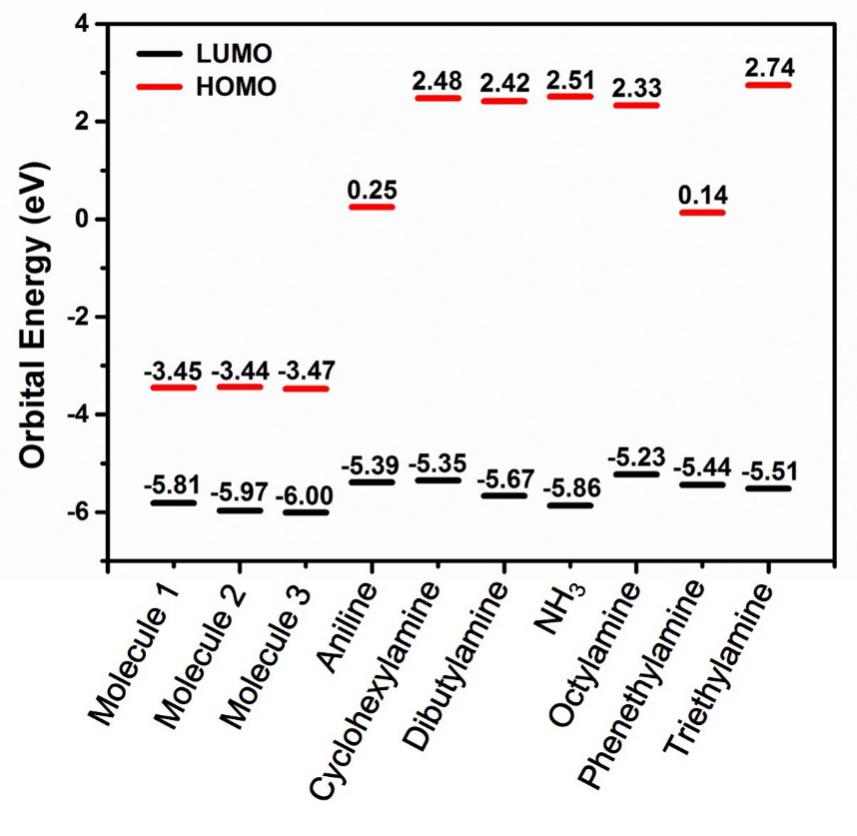


Fig. [S3]. The calculated LUMO and HOMO energies for molecule **1**, molecule **2**, molecule **3** and amines with geometry optimization by the B3LYP/6-31 G* method.


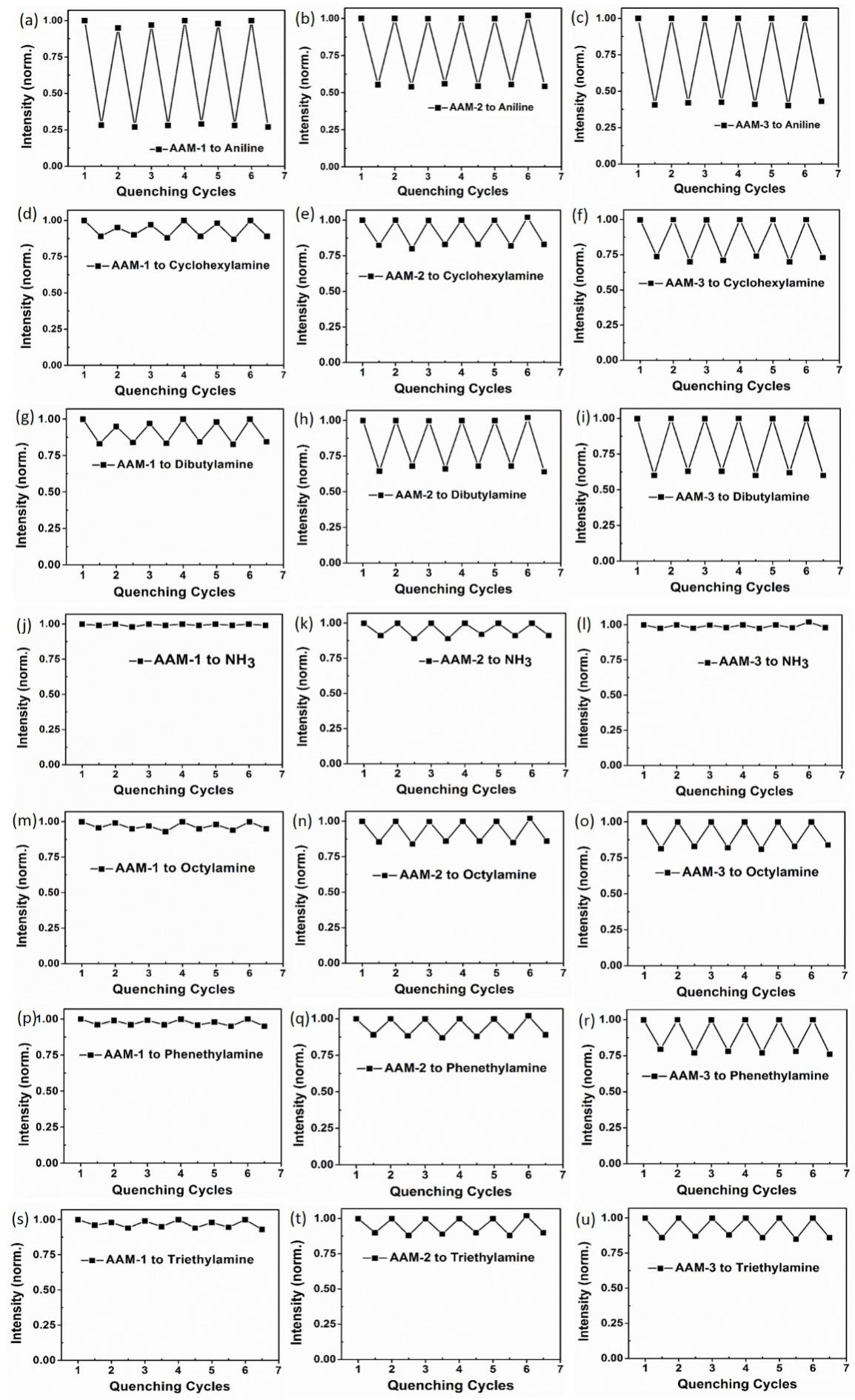


Figure [S4]. Quenching-recovery cycles of PDIs. The fluorescence quenching was performed upon blowing amine vapors (30 ppm). After each cycle of quenching, the fluorescence intensity of the sensory material could be recovered by heating at 60 °C for 1 hour.


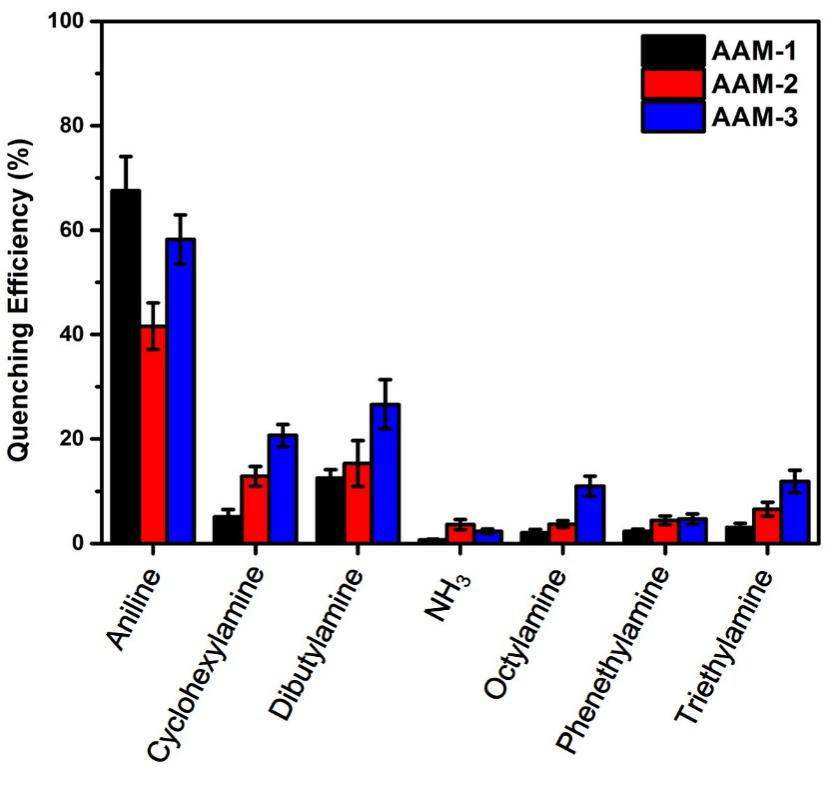


Fig. [S5]. Fluorescence responses of the sensor array constituted by **AAM-1**, **AAM-2** and **AAM-3** to seven selected amine vapors at the concentration of 3 ppm (Error bar is listed on top of each bar in this Figure).


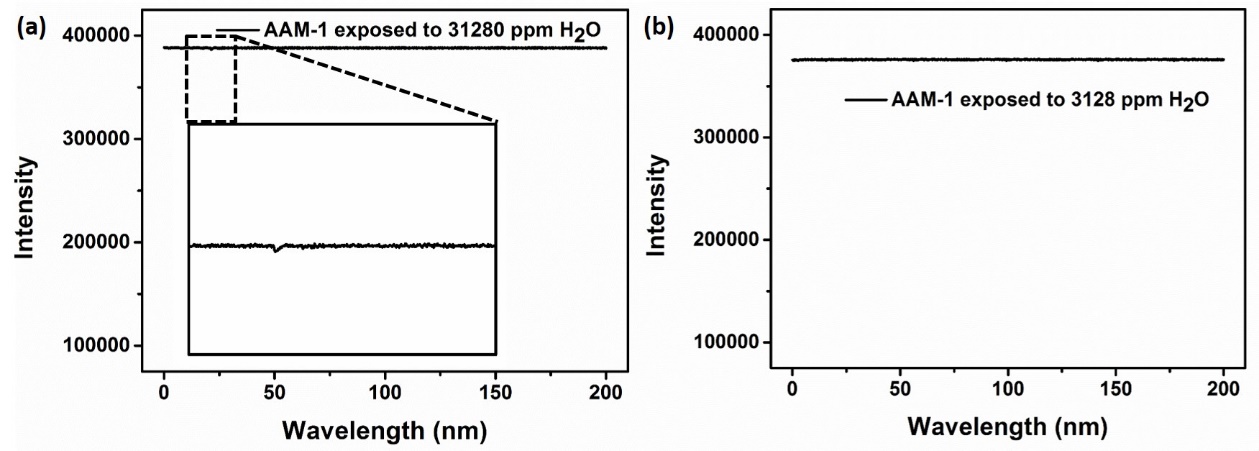


Fig. [S6]. Typical time-dependent fluorescence quenching spectra of **AAM-1** upon exposure to H2O vapor: (a) 31280 ppm; (b) 3128 ppm.


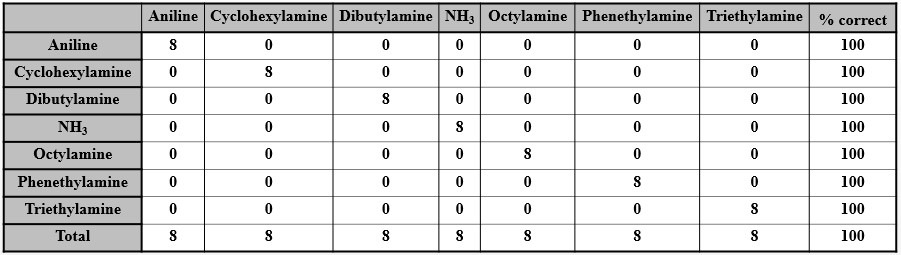


Fig. [S7]. Classification matrix (cases in rows categories, classified into columns) for a LDA using data recorded by the sensor array constituted by **AAM-1**, **AAM-2** and **AAM-3**.


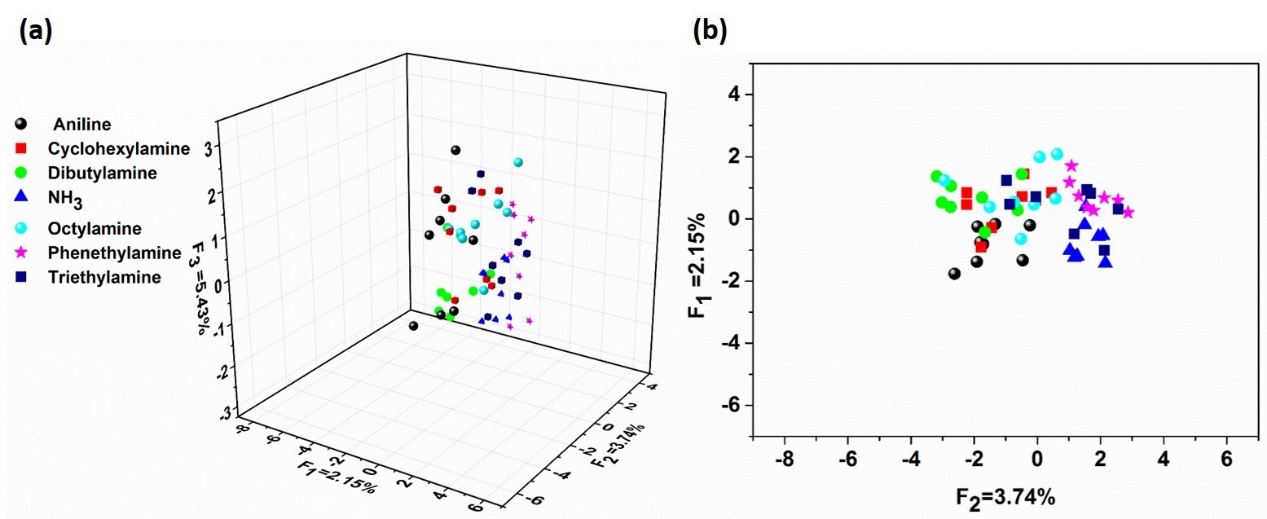


Figure [S8]. Discriminant analysis of a fluorescent sensor array composed of aggregates of AAM-1, AAM-2 and AAM-3. (a) Three-dimensional LDA graph and (b) two-dimensional LDA graph displaying the distinct clustering of seven selected amines (3 ppm).


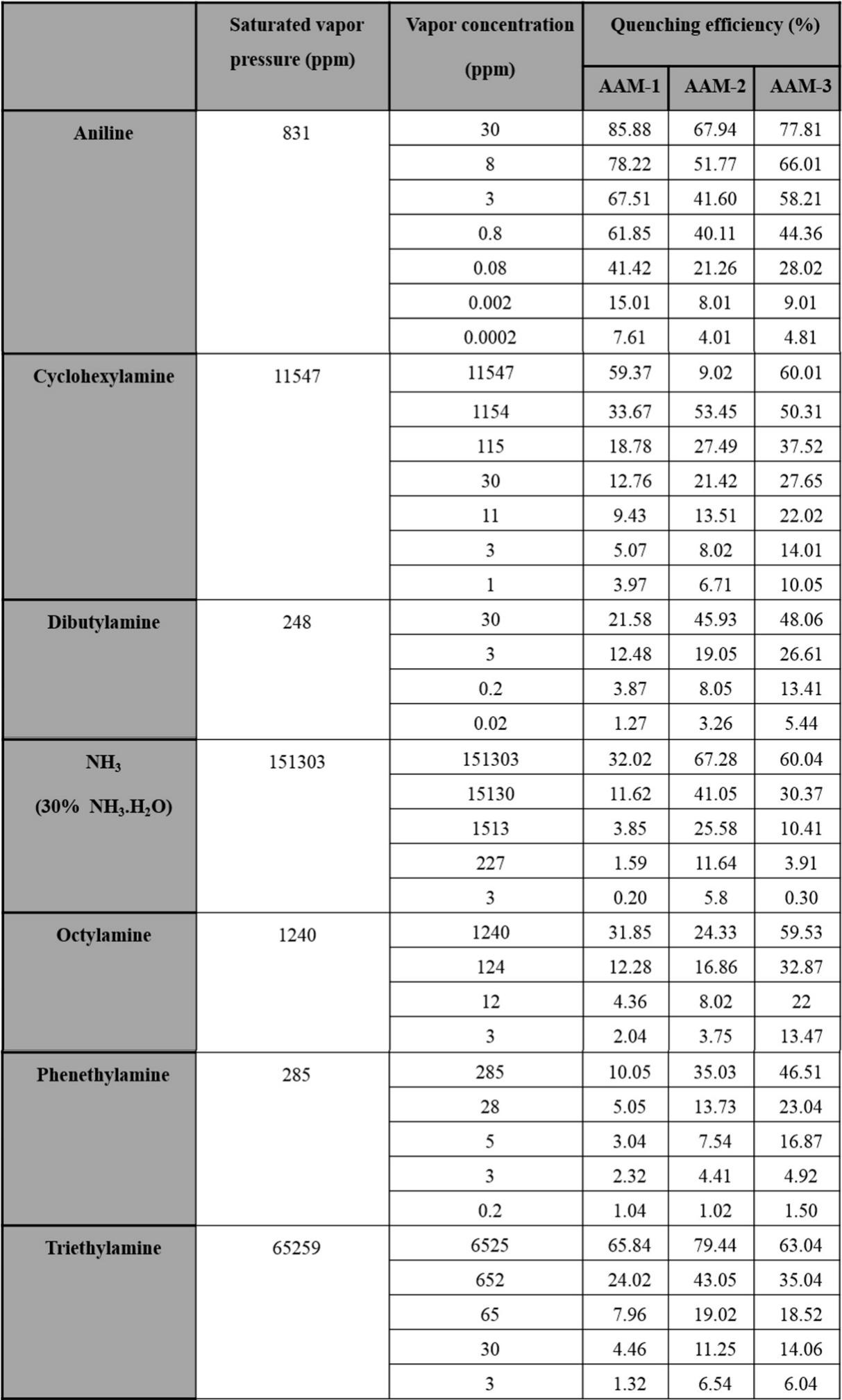


Fig. [S9] The fluorescence response of the sensor array members to amines at different concentrations.


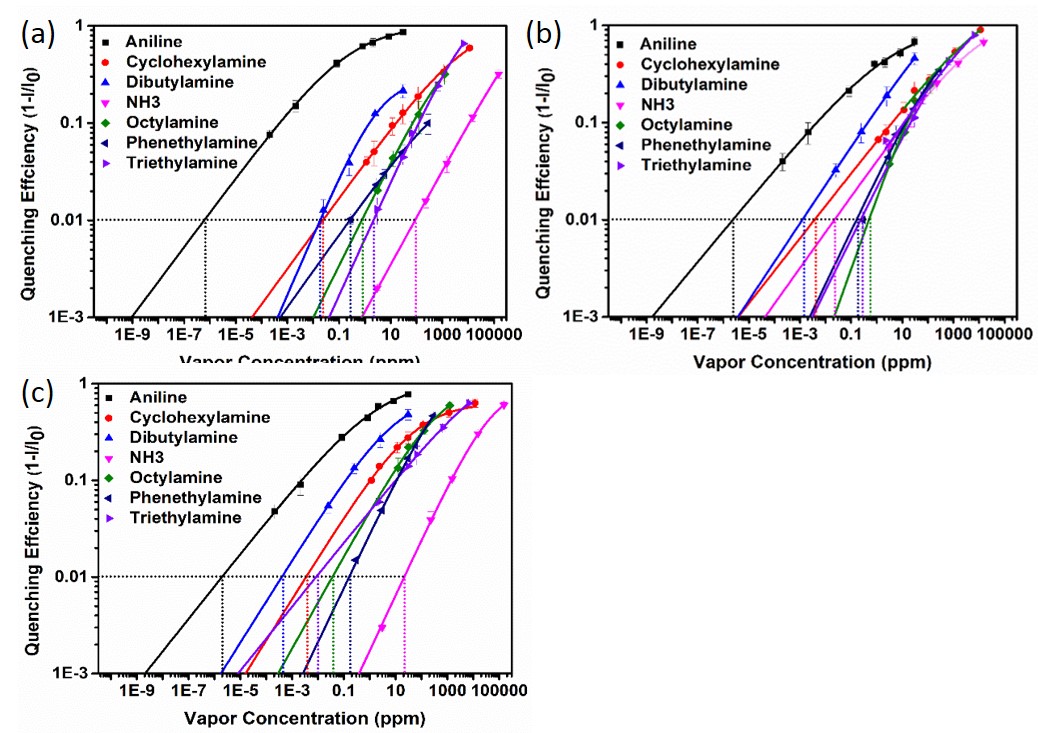


Fig. [S10]. Fluorescence quenching efficiency (1-I/Io) of the sensor array composed by aggregates (a) **AAM-1**, (b) **AAM-2** and (c) **AAM-3** upon selected amine vapors at different concentration.


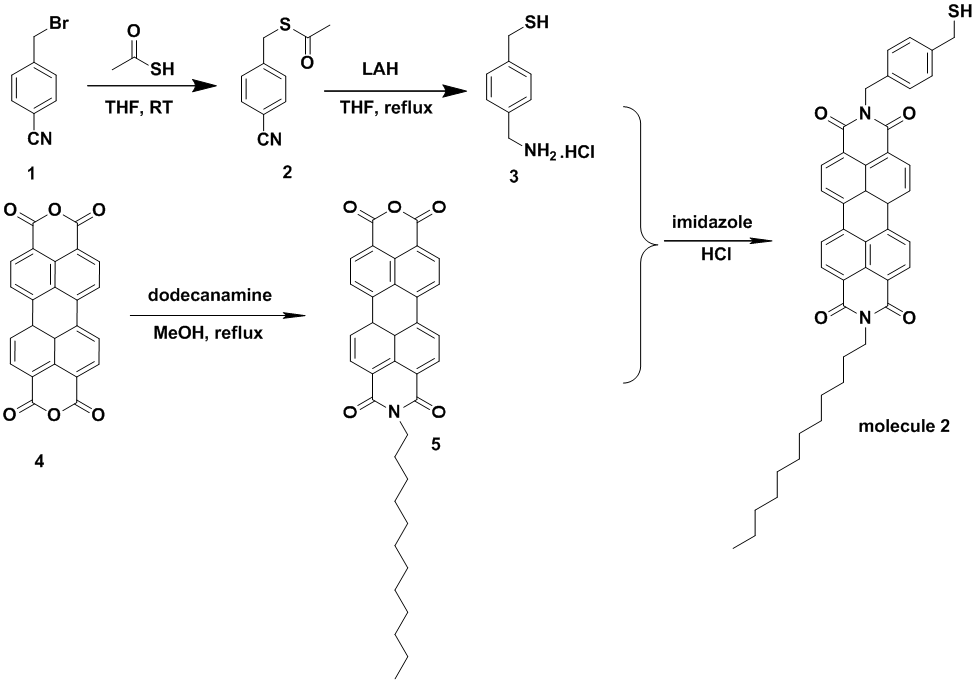


Fig. [S11]. Synthesis procedure of **molecule 2**.


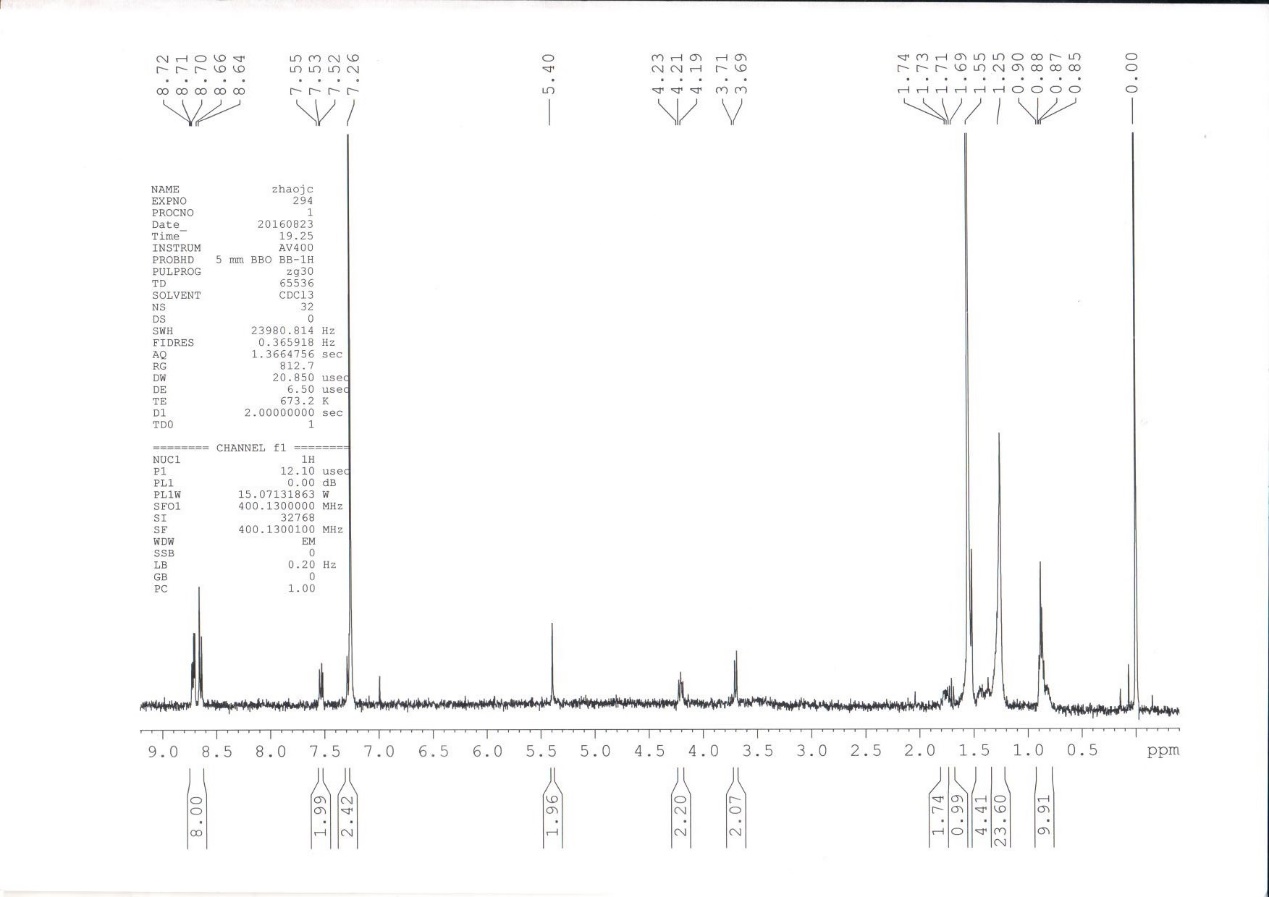


Fig. [S12]. ^1^HNMR of **molecule 2**.


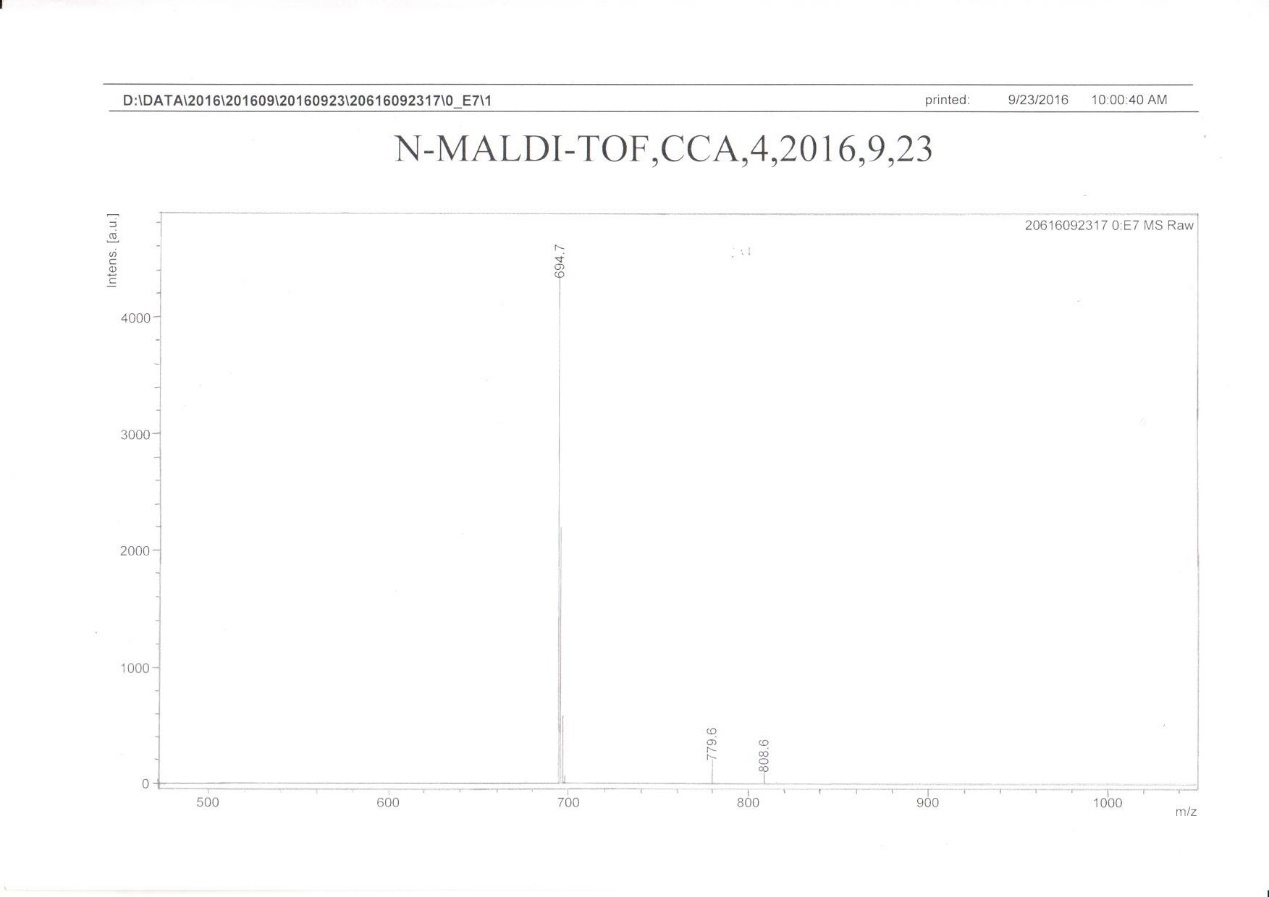


Fig. [S13]. MALDI-MS of **molecule 2**.


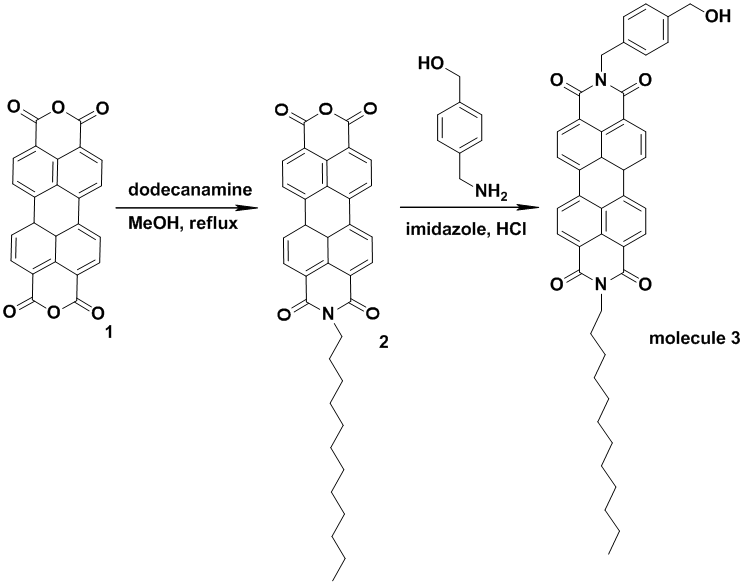


Fig. [S14]. Synthesis procedure of **molecule 3**.


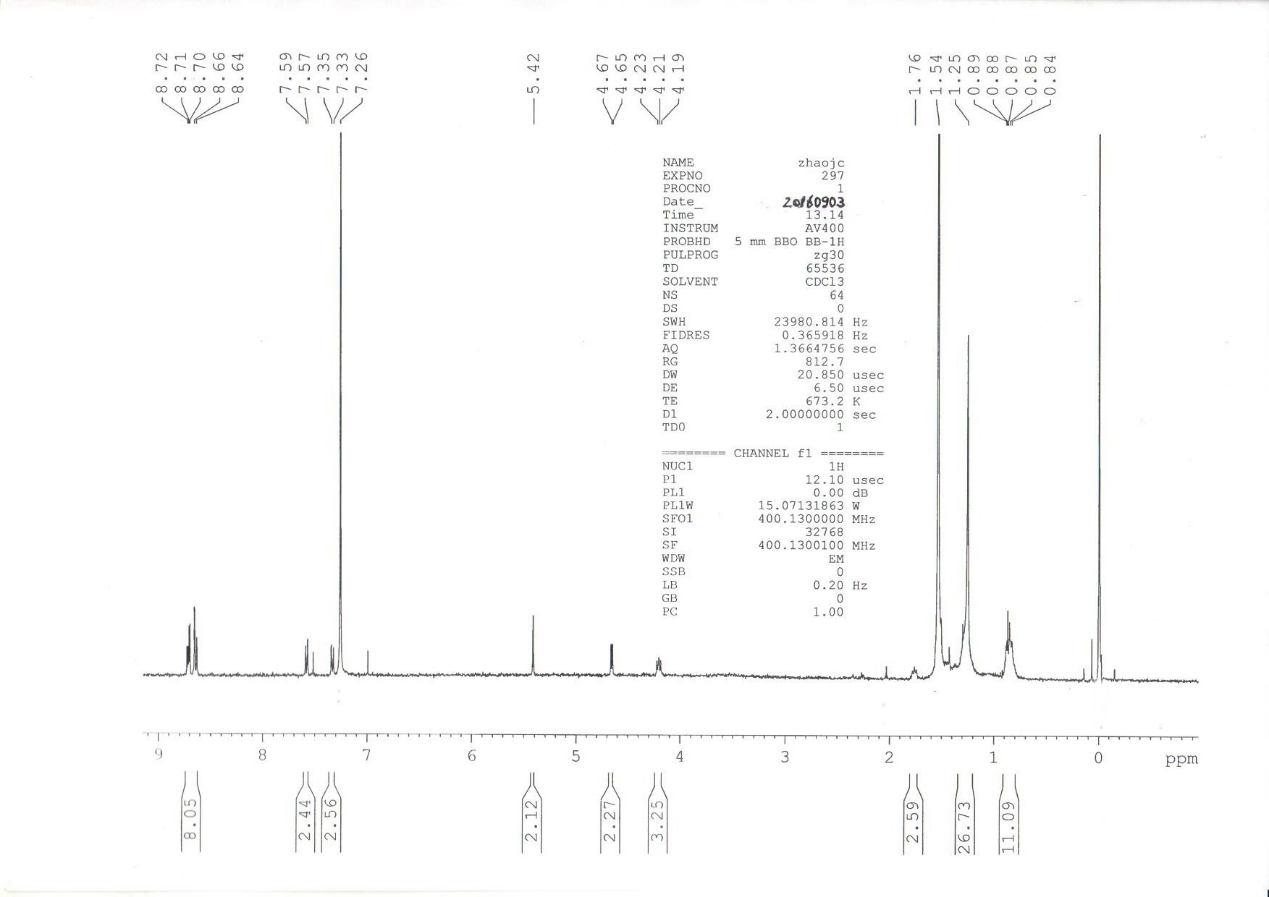


Fig. [S15]. ^1^HNMR of **molecule 3**.


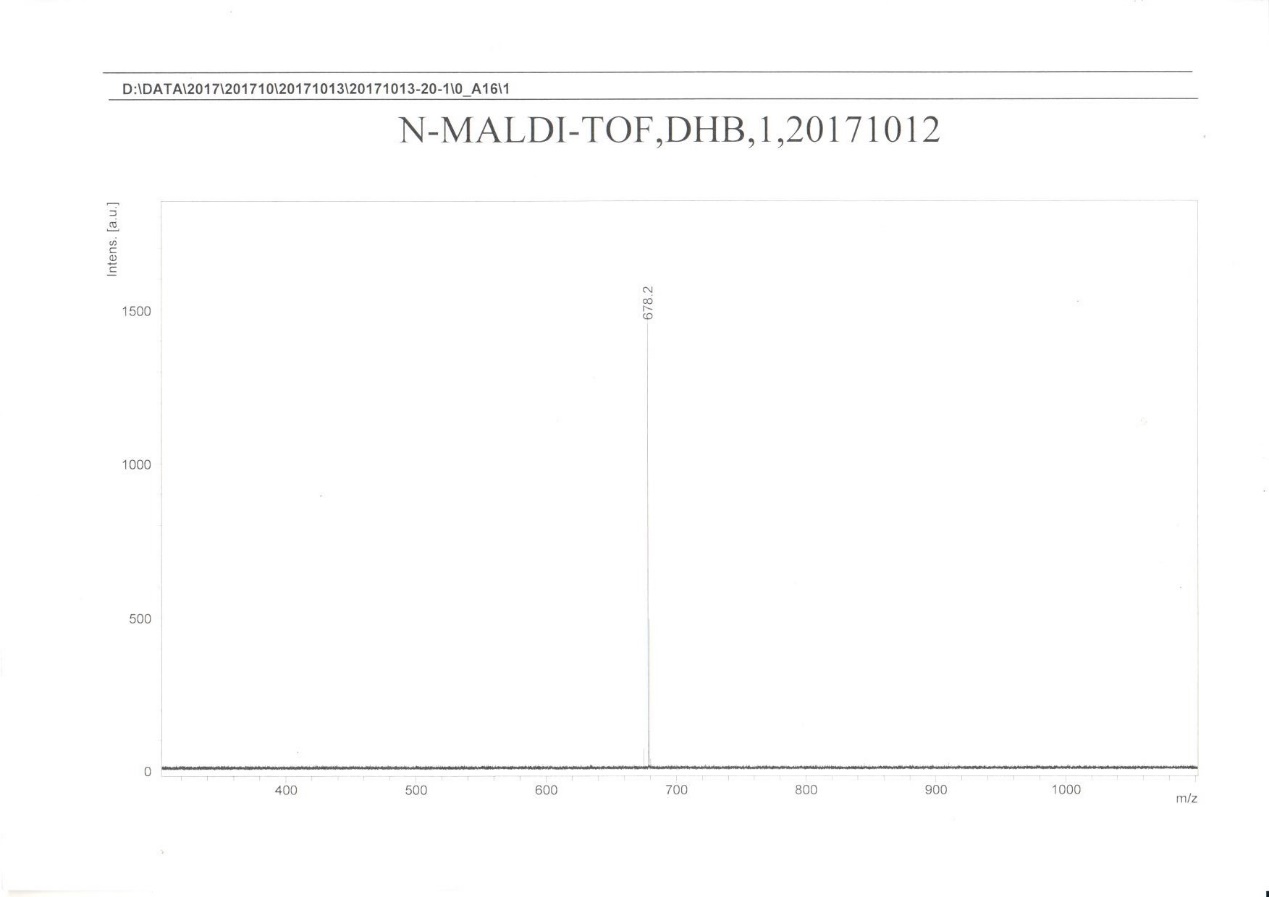


Fig. [S16]. MALDI-MS of **molecule 3**.
